# Échec de la campagne d’éradication de la poliomyélite avec le vaccin oral: on ne vaccine pas sans adhésion

**DOI:** 10.48327/mtsi.v3i4.2023.402

**Published:** 2023-11-16

**Authors:** Bernard SEYTRE

**Affiliations:** bnscommunication, 7 rue Ledion, 75014 Paris

**Keywords:** Poliomyélite, Éradication, Vaccin polio oral (VPO), Vaccin polio inactivé (VPI), Vaccination, Résistance, Poliomyelitis, Eradiction, Oral poliovirus vaccine (OPV), Inactivated poliovirus vaccine (IPV), Vaccination, Resistance

## Abstract

Trente-cinq ans après son lancement, l'Initiative mondiale pour l’éradication de la poliomyélite n'a toujours pas atteint son but, originellement fixé à l'an 2000. Non seulement le virus sauvage de type 1 de la poliomyélite est toujours endémique dans deux pays, mais une nouvelle épidémie due à des virus dérivés du virus vivant atténué utilisé pour le vaccin oral se propage depuis 2016. Les Journées nationales de vaccination (JNV), au cours desquelles des équipes font du porte-à-porte, tout en vaccinant parfois également dans les rues, ont suscité de violentes oppositions particulièrement dans le nord du Nigéria et dans la zone Inde, Pakistan, Afghanistan. Dans les deux régions, la même rumeur s'est développée selon laquelle le vaccin contiendrait des produits stérilisants, pour limiter la population musulmane. Les organisateurs de la campagne ont multiplié en vain les JNV pensant finir par venir à bout des résistances, mais celles-ci ont laissé des poches de population insuffisamment vaccinée, ce qui a permis au virus sauvage de demeurer endémique et à la nouvelle épidémie de virus dérivés du vaccin de progresser. Nous pouvons nous poser la question de ce que la campagne serait devenue si ses organisateurs avaient pris le temps de la réflexion et réorienté leur stratégie pour s'appuyer sur la vaccination de routine du Programme élargi de vaccination, qui ne suscite pas de telles oppositions.

## Abstract (en)

Thirty-five years after its launch, the Global Polio Eradication Initiative has yet to reach its original goal of 2000. Not only is the wild type 1 polio virus still endemic in two countries, but a new outbreak due to viruses derived from the live attenuated virus used for the oral vaccine has been spreading since 2016. The National Immunization Days (NID), during which teams go door-to-door and attract children to be vaccinated, have provoked violent opposition particularly in Northern Nigeria and in the area of India, Pakistan, Afghanistan. In both regions, the same rumor has developed that the vaccine contains sterilizing products, in order to limit the Muslim population. The organizers of the campaign multiplied in vain the NIDs to overcome the resistance, but pockets of insufficiently vaccinated population have persisted. This has allowed the wild virus to remain endemic and the new outbreak of vaccine-derived viruses to progress. We can wonder what the campaign would have become if its organizers had taken the time to reflect and reorient their strategy to rely on the routine vaccination of the Expanded Program on Immunization that does not arouse such opposition.

## Introduction

En cette époque où la rapidité d'une publication entre en compétition avec sa qualité, où l'on diffuse en ligne des *preprints* non relus, la publication récente par Martin Schlumberger, dans *Médecine Tropicale et Santé Internationale,* d'un travail réalisé en 1988 pourrait paraître incongrue [32]. Elle est en fait pleinement d'actualité et vient ajouter un élément troublant aux discussions sur la conduite de la campagne mondiale d’éradication de la poliomyélite dont l’échec est désormais patent. Troublant car, explique Pierre Saliou dans un éditorial, « l'UNICEF avait en son temps opposé un veto formel à cette publication [31] ».

L'année au cours de laquelle Schlumberger et son équipe ont mené leur travail, 1988, n'est pas anodine. Le 13 mai, l'Assemblée mondiale de la santé avait lancé l'Initiative mondiale pour l’éradication de la poliomyélite (Global Polio Eradication Initiative, GPEI) dont l'objectif était d’éradiquer la maladie avant l'an 2000. Leur travail mettait en cause la doxa genevoise qui misait sur des Journées nationales de vaccination (JNV) utilisant le vaccin oral pour vacciner les enfants des pays en développement. L'avantage du vaccin oral, qui utilise un virus vivant atténué, était que, dans des régions aux conditions sanitaires médiocres, lorsqu'il était administré à un certain nombre d'enfants il vaccinait leur entourage incognito en se transmettant spontanément d'un enfant à l'autre.

Las, au tournant du siècle la poliomyélite n'avait pas disparu. L’éradication fut repoussée à 2004, 2008, 2012, 2018 et finalement 2026, objectif qui, en 2023, paraît hors de portée. Dans un article récent, le directeur de la GPEI s'est félicité que « le nombre de cas dans le monde a diminué de plus de 99% depuis 1988 »… reprenant mot pour mot ce que l'Organisation mondiale de la santé (OMS) avançait déjà en 2000 comme preuve de la réussite de sa stratégie [24].

Pourquoi les moins de 1% de cas restants demeurent-ils hors d'atteinte depuis 22 ans? Des analyses portant sur des aspects biologiques ou virologiques ont été publiées ces dernières années [4]. Mais si, comme l'article de Schlumberger amène à se le demander, le choix considéré comme indiscutable de vaccinations massives avec le vaccin oral n'avait pas été le bon?

## Une stratégie qui ne correspondait pas aux réalités locales

Après le succès de la campagne d’éradication de la poliomyélite en Amérique du Sud, l'OMS décida en 1988 de déployer la même stratégie dans l'ensemble des pays en développement: cibler les jeunes enfants avec le vaccin antipoliomyélitique oral (VPO) par une vaccination de masse pendant des JNV [1]. Les souvenirs d’épidémies de poliomyélite dévastatrices chez les adultes, avec leurs alignements de poumons d'acier ventilant des malades, étaient encore vivaces dans les pays industrialisés, ainsi que le formidable espoir suscité par les vaccins et le succès des campagnes de vaccination à la fin des années cinquante.

La situation était cependant différente dans les pays en développement, où la poliomyélite était une maladie infantile avec des complications limitées et relativement rares. Selon l'OMS, il y avait environ 350 000 cas de poliomyélite dans le monde en 1988. La même année, le nombre de décès d'enfants de moins de 5 ans attribuables à diverses maladies était de 600 000 au Pakistan, 820 000 au Nigéria et 3,6 millions en Inde, pour ne citer que quelques exemples [28].

Comment les populations de ces pays étaientelles censées comprendre le slogan de la GPEI « Chasser la polio de [suivi du nom du pays] » pour une maladie souvent inconnue [19]? Privés d'accès aux structures sanitaires, bénéficiant rarement de la médecine moderne, comment les habitants des communautés les plus pauvres de ces pays pouvaient-ils percevoir des JNV pendant lesquelles des équipes de vaccination utilisaient les moyens les plus bruyants, voyants et agressifs – haut-parleurs, tambours, musique, banderoles, éléphants dans les pays asiatiques – pour attirer les enfants et leur offrir un produit médical gratuit, fourni par leur gouvernement avec l'aide de pays occidentaux? Comment pouvaientils comprendre qu'on fasse du porte-à-porte pour dénicher et vacciner leurs enfants, alors que peu d'efforts étaient entrepris contre des maladies omniprésentes ou, simplement, pour leur fournir de l'eau potable? Comme l'a relevé un article de *PLoS Medicine* à propos des JNV au Nigéria en 2007, c’était « aussi inhabituel qu'un étranger qui ferait du porte-à-porte en Amérique pour distribuer des billets de 100 dollars [16] ». « Pourquoi ne vont-ils pas à l'hôpital pour aider les malades, au lieu de s'occuper seulement de la polio? » et « Pourquoi insistent-ils uniquement sur la polio? » étaient parmi les questions entendues par des équipes de vaccination [29].

La mise en œuvre de cette stratégie difficilement compréhensible par des populations déshéritées s'est en outre accompagnée d'attitudes qui ne pouvaient que les heurter. Une pratique courante, par exemple, consistait à ce que des équipes préparatoires visitent les maisons pour compter le nombre d'enfants et inscrire ce nombre sur leur porte. Ce qui était susceptible d’être perçu comme un manque de respect de l'intimité dans toute région du monde a été interprété comme « la marque de la bête » du livre de l'Apocalypse parmi les Kitawalas, en République démocratique du Congo (RDC) [39].

Il n'est donc guère surprenant que dès leur lancement, les JNV aient suscité des résistances dans des pays africains et asiatiques. À une époque où Internet ne relayait pas encore les rumeurs à l’échelle mondiale, la même croyance est apparue sur les deux continents selon laquelle le VPO contenait des produits pour stériliser les enfants afin de limiter la population [6]. Dans la plupart des pays, l'ampleur des refus resta limitée et la répétition des JNV finit par éliminer les cas de poliomyélite en quelques années. Cependant, dans des pays divisés par des conflits politiques, ethniques ou religieux tels que le Nigéria, l'Inde, le Pakistan et l'Afghanistan, ces refus se sont transformés en une vague d'hostilité, avec un rejet massif de la vaccination qui est allé jusqu’à l'assassinat d’équipes de vaccinateurs, dont 31 ont, par exemple, été tués au Nigéria et au Pakistan en 6 mois, en 2012 [39].

En 2003, trois États du nord du Nigéria ont interdit la vaccination contre la poliomyélite, avec des explications telles que « Depuis le 11 septembre, le monde musulman se méfie de toute initiative du monde occidental; les vaccins contre la poliomyélite inquiètent beaucoup notre population » et « Nous croyons que des Hitler des temps modernes ont délibérément introduit des produits stérilisants dans les vaccins oraux contre la poliomyélite [22] ». L'UNICEF et l'OMS ont associé des leaders traditionnels et religieux à la GPEI, organisant par exemple des voyages sur des sites de production de VPO dans un pays musulman, l'Indonésie, qui livrait les vaccins pour leurs propres pays, ou encore une réunion de 150 responsables musulmans et chefs traditionnels de sept pays d'Afrique centrale et occidentale dans le nord du Nigéria [16]. Ces initiatives ont permis de vaincre les réticences des responsables religieux et traditionnels, si bien que la vaccination a repris en 2004 dans les trois États nigérians. Les refus de vaccination ont cependant persisté et de nouveaux cas causés par le virus de la poliomyélite sauvage ont été détectés jusqu'en 2016 au Nigéria, avec un maximum de 798 en 2008.

Un scénario très proche se déroulait parallèlement dans la région Inde/Pakistan/Afghanistan. La résistance à la vaccination s'est développée dans les communautés musulmanes pauvres, renforcée en Inde par le souvenir de la campagne de masse menée par le gouvernement en 1976 pour stériliser les hommes et un sentiment d’être victimes d'injustices de la part des intégristes hindouistes. Le gouvernement indien, dirigé par des partis hindouistes, était soupçonné d'utiliser le VPO pour stériliser les enfants, avec le soutien américain. En 2003, le directeur de la GPEI reconnaissait ainsi qu'en Inde « une proportion importante d'enfants n'a jamais été immunisée, principalement dans les communautés musulmanes, […] nous devons surmonter un siècle de méfiance [34] ». En 2005, le Groupe consultatif d'experts sur l'Inde de la GPEI estimait que les « jeunes enfants et les musulmans » étaient des « groupes minoritaires à risque élevé [14] ». Là aussi, l'UNICEF et l'OMS ont associé les responsables musulmans et organisé des visites de sites de production du vaccin dans des pays musulmans. Mais, comme au Nigèria, la résistance à la campagne de vaccination a persisté dans les communautés musulmanes, en particulier dans les États indiens de l'Uttar Pradesh et du Bihar. En 2019, la campagne de vaccination contre la poliomyélite a même dû être suspendue pendant plusieurs mois après qu'une foule ait attaqué et incendié un hôpital dans le district de Peshawar, au Pakistan, à la suite d'une rumeur selon laquelle le vaccin aurait rendu malade et tué des enfants [15].

Alors que le dernier cas de poliomyélite causé par le virus sauvage a été signalé en Inde en 2011, la maladie demeure endémique au Pakistan et en Afghanistan, avec 20 cas rapportés en 2022 au Pakistan et 2 en Afghanistan [12]. Parmi les trois types de virus de la poliomyélite, les types 2 et 3 sont considérés comme éradiqués et les cas dus à un virus sauvage au cours des dernières années sont causés par le type 1. Si l'on se fonde sur l'estimation selon laquelle 200 enfants sont infectés pour 1 cas de paralysie, 4 000 et 400 enfants ont été porteurs du virus, respectivement, au Pakistan et en Afghanistan en 2022 [3]. Cette année-là, le virus a été réintroduit depuis le Pakistan au Mozambique, où il avait été éliminé 30 ans plus tôt [10].

Il faut souligner que les refus massifs de vaccination ne sont pas liés directement à la religion musulmane, la GPEI n'ayant pas rencontré de difficultés significatives pour obtenir l'adhésion des populations dans des pays principalement musulmans, comme ceux d'Afrique du Nord et du Moyen-Orient. Le refus massif du vaccin est concentré dans des « zones de conflit intense » et apparaît comme la conjonction de la pauvreté et d'une méfiance ancienne envers le pouvoir et éventuellement, derrière lui, l'Amérique et Israël [35].

Parallèlement à la persistance de l'endémie de virus sauvage de type 1, plusieurs épidémies dues à des virus dérivés des vaccins sont apparues. En effet, le virus vivant atténué du vaccin oral utilisé pour la vaccination de masse retourne à la virulence et cause la poliomyélite, en moyenne une fois pour 2,6 millions de doses utilisées [23]. Si plusieurs foyers ont pu être éliminés, des virus dérivés du vaccin *(circulating vaccine-derived poliovirus,* cVDPV) se propagent dans le monde depuis 2016. En 2021, 698 cas de poliomyélite et détections de cVDPV dans l'environnement ont été rapportés dans 36 pays [27]. Les cVDPV sont surtout de type 2 (cVDPV2) [4].

**Figure 1:**
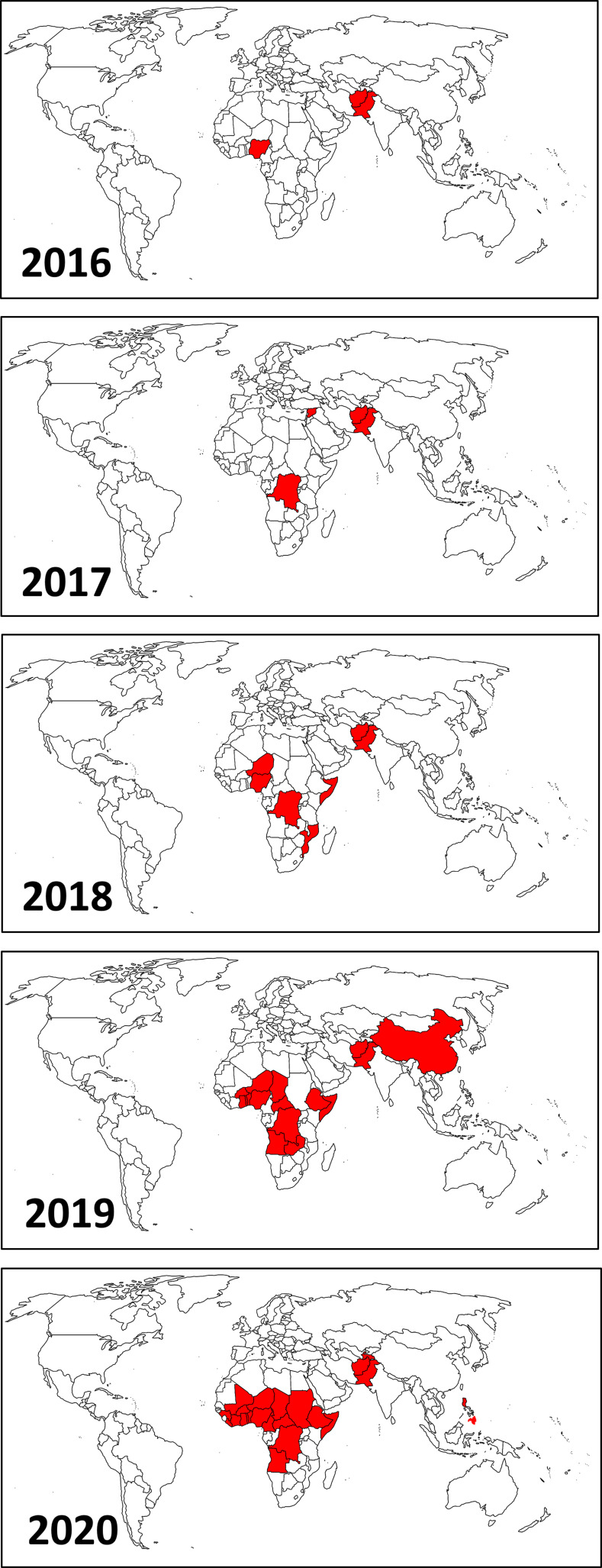
Pays touchés par le virus sauvage de type 1, ou un cVDPV2, ou les deux. Countries affected by type 1 wild poliovirus, or cVDPV2, or both.

L’épidémie de cVDPV est apparue dans les deux régions du monde marquées par de fortes résistances à la vaccination d'où elle s'est étendue (Fig. 1). Deux cas de poliomyélite dus au cVDPV2 ont été signalés en 2016 au Pakistan et au Nigéria [4]. En 2017, des cas ont été signalés en RDC ainsi qu'en Syrie où l’épidémie a été interrompue par la vaccination. En 2018, 64 des 71 cas signalés se trouvaient en RDC, au Nigéria et au Niger, où l’épidémie n'a pas encore été maîtrisée. À partir de 2019, l’épidémie s'est propagée dans le monde entier pour atteindre Israël, le Royaume-Uni et les États-Unis, où elle a été détectée dans les eaux usées et a causé 1 cas de poliomyélite dans l’État de New York en juillet 2022. Les pays touchés par la nouvelle épidémie au cours des trois premières années sont ceux où le virus sauvage est toujours en circulation (Pakistan, Afghanistan) ou l’était jusqu'en 2011 (Nigéria, RDC) ou 2012 (Niger). En comparaison, les pays où le virus sauvage a été éliminé en quelques années de campagnes de vaccination de masse sont restés exempts de l’épidémie de cVDPV jusqu'en 2019 [27].

Le retard ou l’échec de l’élimination de la poliomyélite dans les pays où la GPEI a rencontré un niveau élevé de refus a eu deux conséquences. Premièrement, la répétition des campagnes de vaccination avec le vaccin oral pendant 10, voire 20 ans, avec une moyenne d'un milliard de doses utilisées dans le monde pendant au moins 10 ans, a multiplié les risques d'apparition de cVDPV [9]. En 2005, les enfants de moins de 5 ans vaccinés ont reçu en moyenne 15 doses de VPO dans les États indiens de l'Uttar Pradesh et du Bihar [13]. Deuxièmement, les refus de vaccination ont créé des poches d'enfants non immunisés qui ont favorisé la propagation des nouveaux virus pathogènes dérivés du vaccin oral [5, 21].

L'insuffisance d'une couverture vaccinale peut être multifactorielle. Elle peut être due à une mauvaise gestion de la vaccination, des difficultés logistiques, un manque d'approvisionnement en vaccins ou des conflits armés, ethniques ou politiques. Cependant, la GPEI a su résoudre ce type de difficultés dans différentes régions du monde et les résistances à la vaccination semblent donc être le facteur déterminant de l'insuffisance de la couverture vaccinale dans certaines populations, ce qui a permis à la fois la persistance de la circulation du virus sauvage et l’émergence de l’épidémie de cVDPV [18].

## Des réponses tardives

En 2010, Larson et Heymann ont estimé à propos de la campagne de vaccination contre la poliomyélite au Nigéria: « La crise aurait pu être évitée grâce à un effort beaucoup plus précoce pour mobiliser les communautés et renforcer la confiance dans des domaines où les niveaux globaux de méfiance étaient bien connus [20]. » L'histoire ne manque pas d'exemples de résistances à des campagnes de vaccination, non pas en raison d'effets secondaires réels des vaccins, mais à cause des représentations de la population sur les autorités qui promouvaient cette vaccination. Une telle résistance a même été décrite au Nigéria dans les années du lancement de la campagne pour l’éradication de la poliomyélite dans le pays. Un article publié en 1996 décrivait comment le Rotary International venait d'annuler un projet de vaccination dans le nord du Nigéria parce que les parents refusaient de faire vacciner leurs enfants [30]. Les auteurs rapportaient des rumeurs selon lesquelles des produits stérilisants étaient introduits dans des vaccins… comme dans les bouillons Knorr vantés par des publicités. Ils écrivaient: « Si elle n'est pas nouvelle au Nigéria, la méfiance envers les programmes de planning familial est en progression. Cette forte méfiance a des implications pour des programmes de santé actuels soutenus par des organisations non gouvernementales américaines et les Nations unies dans le nord du Nigéria. » L'article décrivait la perception du déclin des services de santé alors que les programmes de planning familial étaient renforcés, le sentiment d’être victimes d'inégalités économiques, l'impact local des enjeux politiques nigérians et l'hostilité à la politique américaine au Nigéria et au Moyen-Orient, les Américains étant soupçonnés de vouloir limiter la population musulmane. Le Rotary International était, et est toujours, l'un des principaux partenaires de la GPEI. Les JNV utilisant le vaccin oral ont commencé au Nigéria l'année même de la publication de cet article décrivant une situation qui allait être le terreau des résistances à la campagne d’éradication de la poliomyélite.

Les responsables de la campagne sur le terrain n'ont, bien sûr, pas ignoré les résistances auxquelles ils se heurtaient. Dès 1999, des rapports de la GPEI mentionnèrent les rumeurs à l'origine de refus massifs de vaccination et recommandèrent d'associer les dirigeants communautaires et religieux locaux [25, 26]. Dans les pays déchirés par des conflits ethniques, religieux ou politiques, des mesures auraient pu être prises dès les premières résistances pour identifier les facteurs de refus, étudier les perceptions de la campagne de vaccination dans les communautés et élaborer un programme visant à gagner l'adhésion, d'abord, des dirigeants traditionnels et religieux locaux, puis, en partie à travers eux mais pas seulement, celle de la population. Comme l'ont noté les auteurs d'une analyse récente de la campagne d’éradication de la poliomyélite en RDC et en Éthiopie: « Les études socio-anthropologiques et les évaluations du système de santé sont des précurseurs importants de la mise en œuvre d'un programme pour identifier les obstacles potentiels à cette mise en œuvre, liés à la méfiance des communautés, aux enjeux politiques locaux et nationaux, à la gouvernance et à la responsabilisation [6]. »

Plutôt que de marquer une pause pour adapter sa stratégie, la GPEI a tenté de passer en force, multipliant des JNV qui ne faisaient que renforcer la méfiance de certaines catégories de la population. Il fallut des années pour s'attaquer réellement aux facteurs de refus de la vaccination. Selon l'UNICEF, la « stratégie d'inclusion a commencé à prendre de l'ampleur en 2003 » en Inde et des organisations musulmanes ont été approchées en 2004 [37]. Dans un rapport de 2011, 16 ans après les premières JNV, l'UNICEF notait que « l'engagement au niveau communautaire commence à donner des résultats », mentionnant un « partenariat » au Pakistan qui « est un début très prometteur et crucial pour élargir le soutien local » et un « programme » dans le nord du Nigéria qui « teste plusieurs approches de mobilisation appuyées sur les remontées des communautés [40] ». Le même rapport mentionne toujours des « poches de refus » au Nigéria, en RDC, au Pakistan, en Afghanistan et au Tchad. Au Nigéria, 18 000 imams ont reçu une formation et des informations sur la poliomyélite entre 2010 et 2014, quatorze ans après les premières JNV et l'article cité précédemment qui exposait la problématique des rumeurs [11].

Les dirigeants musulmans locaux des communautés réticentes ont finalement été convaincus de l'innocuité et de l'efficacité du VPO et sont devenus des promoteurs de la vaccination. Cependant, il est plus facile de perdre la confiance que de la regagner, et la méfiance suscitée pendant plus de dix ans dans la population n'a jamais complètement disparu. Elle est aujourd'hui utilisée par des mouvements radicaux comme Boko Haram au Nigéria ou les talibans au Pakistan et en Afghanistan. Ces derniers n'ont, en outre, pas manqué d'exploiter le recours de la CIA à une fausse campagne de vaccination pour localiser Ben Laden grâce à l'ADN de ses enfants prélevé sur les aiguilles des seringues, en 2011.

## Une autre stratégie de vaccination était-elle possible?

Dans un article récent, Jacob John *et al.* estiment que plus de 20 000 enfants ont souffert de poliomyélite à cause de l'utilisation du VPO et demandent aux responsables de la GPEI d'abandonner ce vaccin pour le remplacer exclusivement par le vaccin injectable (vaccin polio inactivé, VPI) qui n'a jamais provoqué aucun cas de poliomyélite, et ce malgré la facilité d'administration du vaccin oral [17]. À la lecture de l'article de Schlumberger, on peut se demander si la stratégie des bruyantes Journées nationales de vaccination qui visent à administrer le vaccin oral au maximum d'enfants attirés sur des places publiques ou débusqués au porte-à-porte était la seule possible. Dans leur travail mené en 1988 mais interdit alors de publication par l'UNICEF, les auteurs montrent en effet que l'administration du VPI en même temps que d'autres vaccins du Programme élargi de vaccination (PEV) donne des résultats de couverture vaccinale équivalents, pour un coût inférieur. Or, loin de susciter des résistances, la vaccination dans le cadre du PEV est bien acceptée, y compris dans les régions mentionnées précédemment où la couverture vaccinale contre la poliomyélite est particulièrement faible, avec les conséquences que nous avons vues. Par exemple, la couverture vaccinale contre la rougeole dans l’État nigérian de Kano, où la résistance à la campagne d’éradication de la poliomyélite a été particulièrement forte, a été de 95% en moyenne chaque année de 2008 à 2014 [2]. En 2021, la couverture par le vaccin DTP1 (diphtérie, tétanos, coqueluche) était de 90% au Pakistan et de 93% en Afghanistan, malgré la guerre [36, 38]. Ces taux sont peut-être surestimés à cause d'une sous-estimation de la population d'enfants dont beaucoup ne sont pas déclarés à l’état civil, mais la couverture réelle est cependant incontestablement très élevée.

Nous pouvons ainsi nous poser la question: que se serait-il passé dans les régions où les résistances mettaient la campagne d’éradication en échec si ses organisateurs avaient décidé de changer de stratégie, de supprimer les JNV, d'intégrer le VPI au PEV et de renforcer cette vaccination pour qu'elle atteigne toutes les communautés?

## Conclusion

La campagne d’éradication de la poliomyélite est manifestement un échec et le dernier objectif en date, 2026, semble aujourd'hui inatteignable. Si elle devient un jour une réalité, l’éradication aura 30 ans de retard en ayant coûté des sommes astronomiques. À son lancement, les organisateurs de la campagne étaient forts du fait qu'avec l'absence de réservoir animal et l'utilisation de vaccins complémentaires, les conditions biologiques de l’éradication étaient réunies [7]. Si l'on a pu dire qu'il n'y a pas de maladie mais seulement des malades, les vaccins, de même, n'existent que dans la mesure où des personnes sont vaccinées [8].

Alors que des résistances sont apparues dès le début de la campagne d’éradication, gagner l'adhésion des populations n’était pas une priorité pour les responsables de cette campagne. Loin de prendre le temps d'analyser ces résistances afin de comprendre pourquoi ce qui avait fonctionné dans certains pays en développement ne fonctionnait pas dans d'autres et d'adapter leurs stratégies de vaccination et de communication, ils ont continué à dérouler le même plan, année après année, ancrant dans certaines communautés une méfiance qui tournait souvent à l'hostilité. Une réorientation de la campagne d’éradication de la poliomyélite aurait probablement évité des années de Journées nationales de vaccination, limitant le risque d'apparition d’épidémies dues à des virus dérivés du vaccin et augmentant les chances d’éradication du virus sauvage.

Vacciner ne consiste pas seulement à fournir les vaccins, la logistique et les ressources financières et humaines. La population doit adhérer à la vaccination. Cette adhésion dépend de l'ensemble des connaissances, perceptions et représentations non seulement sur le vaccin concerné (bénéfices attendus et effets secondaires) mais surtout sur les promoteurs de la vaccination, ce que nous proposons d'appeler la « culture en santé » des populations [33]. Les Sud-Américains ne sont pas les Africains. Les Sénégalais ne sont pas les Nigérians. Et les Nigérians du Nord ne sont pas les Nigérians du Sud.

On ne vaccine pas sans adhésion.

## Conflits d'intérêts

L'auteur ne déclare aucun lien d'intérêts.
